# The inner‐rod component of *Shigella flexneri* type 3 secretion system, MxiI, is involved in the transmission of the secretion activation signal by its interaction with MxiC

**DOI:** 10.1002/mbo3.520

**Published:** 2017-12-01

**Authors:** Nargisse El Hajjami, Simon Moussa, Jonathan Houssa, Daniel Monteyne, David Perez‐Morga, Anne Botteaux

**Affiliations:** ^1^ Laboratoire de Bactériologie Moléculaire Faculté de Médecine Université Libre de Bruxelles Bruxelles Belgium; ^2^ Laboratoire de Parasitologie Moléculaire Faculté des Sciences Université Libre de Bruxelles Charleroi Belgium; ^3^ Center for Microscopy and Molecular Imaging‐CMMI Université Libre de Bruxelles Gosselies Belgium

**Keywords:** needle components, secretion regulation, *Shigella* virulence, T3SS activation signal, type 3 secretion system

## Abstract

The virulence of *Shigella* mainly resides in the use of a Type 3 Secretion System (T3SS) to inject several proteins inside the host cell. Three categories of proteins are hierarchically secreted: (1) the needle components (MxiH and MxiI), (2) the translocator proteins which form a pore (translocon) inside the host cell membrane, and (3) the effectors interfering with the host cell signaling pathways. In the absence of host cell contact, the T3SS is maintained in an “off” state by the presence of a tip complex. We have previously identified a gatekeeper protein, MxiC, which sequesters effectors inside the bacteria probably by interacting with MxiI, the inner‐rod component. Upon cell contact and translocon insertion, a signal is most likely transmitted from the top of the needle to the base, passing through the needle and allowing effectors release. However, the molecular mechanism underlying the transmission of the activation signal through the needle is still poorly understood. In this work, we investigate the role of MxiI in the activation of the T3SS by performing a mutational study. Interestingly we have shown that mutations of a single residue in MxiI (T82) induce an *mxiC*‐like phenotype and prevent the interaction with MxiC. Moreover, we have shown that the L26A mutation significantly reduces T3 secretion. The L26A mutation impairs the interaction between MxiI and Spa40, a keystone component of the switch between needle assembly and translocators secretion. The L26A mutation also sequesters MxiC. All these results highlight the crucial role of MxiI in regulating the secretion and transmitting the activation signal of the T3SS.

## 1 INTRODUCTION

*Shigella* is a highly adapted human pathogen that causes shigellosis also known as bacillary dysentery. This disease is responsible for more than 1 million deaths per year globally, essentially among children under 5 years of age in developing countries (Kotloff, 1999). *Shigella,* like a wide spectrum of gram‐negative bacteria, uses a Type 3 Secretion System (T3SS) to inject virulence proteins into eukaryotic cells (Cornelis, 2006; Galán & Wolf‐Watz, 2006) allowing bacterial entry and dissemination within the gut epithelial lining (Sansonetti, 2006; Schroeder & Hilbi, 2008).

The T3S apparatus (T3SA) is composed of more than 20 proteins assembled into four parts: (1) a cytoplasmic part called the C‐ring, (2) an export apparatus localized in the inner‐membrane ring, (3) a basal body spanning the inner (IM) and outer (OM) membranes, and (4) an extracellular needle (Blocker et al., 1999; Burkinshaw & Strynadka, 2014; Chatterjee, Chaudhury, McShan, Kaur, & de Guzman, 2013). In the case of *Shigella*, this needle is built up by the helical assembly of more than 100 copies of MxiH, a small globular protein mainly composed of two α‐helices (Blocker et al., 2001; Demers et al., 2013; Marlovits et al., 2004). Moreover, a minor needle component, called MxiI, sharing some sequence similarities with MxiH, is probably localized at the base and forms the inner rod of the T3SA between the IM and the OM (Blocker et al., 2001; Marlovits et al., 2004). As sequence similarities exist between T3SA components of different bacteria harboring a T3SS, homologous proteins of MxiI are found in *Yersinia* (YscI), *Salmonella* (PrgJ), *Pseudomonas* (PscI), or *Burkhoderia* (BsaK). Recently, MxiI homologous protein, PrgJ, has been shown to interact with the cytoplasmic part of proteins composing the export apparatus in *Salmonella* (Dietsche et al., 2016).

At 37°C, MxiH and MxiI are the first substrates secreted through the T3SA allowing the needle to reach the length of about 45 nm (Tamano, Aizawa, & Sasakawa, 2002). At that stage, the cytoplasmic part of Spa40 (Spa40_CT_), an inner‐membrane protein, undergoes a conformational change following its autocleavage into two fragments, called Spa40_CC_ and Spa40_CN_ (Botteaux et al., 2010; Deane et al., 2008a; Monjarás Feria, Lefebre, Stierhof, Galán, & Wagner, 2015; Shen, Moriya, Martinez‐Argudo, & Blocker, 2012) which allows its interaction with the needle length ruler, Spa32 (Botteaux, Sani, Kayath, Boekema, & Allaoui, 2008). This key step is the first switch of substrates specificity which allows stopping needle subunits secretion and starting secretion of proteins that form a “tip complex” (TC), IpaD and IpaB, also called translocator proteins. In the absence of host cell contact, the TC maintains the T3SS in an “off” state (Blocker et al., 2008; Ménard, Sansonetti, & Parsot, 1994; Schiavolin et al., 2013), only secreting a small amount of proteins (also called “leakage” or constitutive secretion). After the host cell is sensed by the TC, a pore is formed inside the host cell membrane by two hydrophobic translocators, IpaC and IpaB (Blocker et al., 1999; Olive et al., 2007; Veenendaal et al., 2007). The resulting pore, called “translocon”, allows the injection into the cell cytoplasm of other T3SS substrates (effectors) that will interfere with the host cell signaling pathways. The release of effectors is controlled by a gatekeeper, MxiC, probably located at the base of the needle (Botteaux, Sory, Biskri, Parsot, & Allaoui, 2009; Martinez‐Argudo & Blocker, 2010). Indeed, MxiC, which is also a T3SS substrate, is directly involved in the regulation of effectors release as the *mxiC* mutant exhibits a constitutive (in the absence of induction) secretion of effectors (Botteaux et al., 2009; Cherradi et al., 2013). Moreover, MxiC also plays a role in translocators secretion after T3SS activation probably through its interaction with IpgC, the translocators chaperone, and Spa47, the T3SS ATPase (Cherradi et al., 2013).

To date, the exact mechanism allowing T3SS activation upon cell contact is not well understood but the most highly supported model (allosteric model) highlights the role of the needle subunits (Kenjale et al., 2005). Indeed, some evidence based on mutational studies on MxiH showed that the needle probably transmits the activation signal from the tip of the needle to the base of the T3SA allowing effectors secretion (Kenjale et al., 2005). Indeed, some point mutations in MxiH (K69A, D72A and R83A) totally abolish effectors secretion and lead to an “effector mutant” phenotype. Interestingly, this phenotype can be rescued by inactivation of *mxiC* in these strains (Martinez‐Argudo & Blocker, 2010).

We have previously shown that the inner‐rod component, MxiI, is also implicated in the signal transmission by generating a point mutation in MxiI (Q67A) leading to an “effector mutant” phenotype, which is also rescued by the *mxiC* inactivation (Cherradi et al., 2013). Moreover, we have shown a direct interaction between MxiC and MxiI, showing for the first time a direct link between the needle and the base for signal transmission (Cherradi et al., 2013). On the other hand, we have identified a mutation in MxiC (F206S) disrupting MxiC–MxiI binding and leading to an early secretion of effectors like in the *mxiC* mutant (Botteaux et al., 2009; Cherradi et al., 2013).

In this study, we have undertaken a novel series of point and random mutations within MxiI to analyze its role in signal transmission aiming to find MxiI mutations that can lead to an *mxiC*‐like phenotype. We have demonstrated here that some mutations of MxiI (on T82 residue) affect its interaction with MxiC and lead to exactly the same phenotype than the *mxiC* mutant. Moreover, the charge of the residue seems to play a key role in the secretion control. We have shown that the C‐terminal part of MxiI (74–93 residues), probably located inside the secretion channel, is sufficient for MxiC binding.

## 2 EXPERIMENTAL PROCEDURES

### 2.1 Bacterial strains and growth conditions

*Shigella flexneri* strains were derivatives of the wild‐type strain M90T (serotype 5a) (Sansonetti, Kopecko, & Formal, 1982). *E. coli* Top10 strains were transformed with pSU18, pQE30, or pGEX4T1 derivatives and BL21 (DE3‐Rosetta) were transformed with pET30a. *Shigella* were phenotypically selected on Congo red (CR) agar plates and grown in tryptic soy broth (VWR) at 37°C with the appropriate antibiotics at the following concentrations: zeocin 50 μg/ml, kanamycin 50 μg/ml, streptomycin 100 μg/ml, ampicillin 100 μg/ml, and chloramphenicol 25 μg/ml for *E. coli* strains and 3 μg/ml for *Shigella* strains.

### 2.2 Plasmids construction

Plasmids and primers used in this study are listed in Tables S1 and S2, respectively. Plasmid pSM6 (pSU18‐*mxiI*), used to complement the *mxiI* mutant, was constructed by inserting a *BamH*I/*Xho*I digested PCR fragment, carrying native *mxiI* gene, into the *BamH*I/*Xho*I sites of the low copy vector pSU18 (Bartolome et al., 1991). Directed mutagenesis was carried out according to the procedure of the Quick Change Mutagenesis kit (Stratagene). The use of each primer in PCR creates a restriction site (Table S2) to easily confirm the introduced mutation. Single directed mutagenesis of residues T82A, T82E, T82R, T82K, L26A, Q67E, and Q67A within *mxiI* was also carried out on plasmids pET30a‐MxiI and pGEX4T1‐MxiI. Random point mutations within MxiI were created by error‐prone PCR as described previously (Weir et al., 2013). As already observed in Cherradi et al., 2013, we failed to detect the expression of the wild‐type or the mutated MxiI proteins by Western blot using anti‐MxiI antibodies probably due to the low expression rate from the pSU18 vector.

### 2.3 Proteins preparation and analysis

Crude extracts and culture supernatant of *S. flexneri* strains were prepared and analyzed as previously described (Allaoui, Sansonetti, & Parsot, 1992). Induction with CR was performed by growing bacteria until OD_600_ has reached 2 units, harvesting by centrifugation, suspending in phosphate buffer saline (PBS) containing 200 μg/ml CR, and incubating for 20 min at 37°C. Bacteria were centrifuged at 13,000 *g* for 15 min at RT and proteins present in the supernatant were analyzed by SDS‐PAGE. Western blotting was performed on polyvinylidene fluoride (PVDF) membranes (GE Healthcare) and developed using chemiluminiscence (Clarity, Biorad). Immunodetection was carried out as described by Botteaux et al. (2009) using monoclonal antibodies directed against His6 motif and a series of polyclonal antibodies against IpaB, IpaA, MxiC, Spa32, IcsB, and GST motif (Barzu et al., 1993; Botteaux et al., 2009; Kayath et al., 2010; Magdalena et al., 2002; Tran Van Nhieu, Ben‐Ze'ev, & Sansonetti, 1997).

### 2.4 Protein production and GST pull‐down assay

*E. coli* BL21 (DE3 Rosetta) was transformed with pGEX4T1 or its derivatives expressing, respectively, GST alone or GST fusion proteins and cultured in 100 ml of lysogeny broth (LB) at 37°C. Protein expression was induced with 0.1 mmol/L isopropyl β‐D‐1‐thiogalactopyranoside (IPTG) for 3 hr at 30°C. Bacteria were harvested, suspended in PBS, and then lyses by sonication in presence of 1% Triton X‐100. The lysates were then clarified by centrifugation and the supernatants mixed with glutathione‐Sepharose 4B matrix beads (GE Healthcare) previously equilibrated with PBS buffer during 1 hr at room temperature on a rotor shaker and then washed three times with PBS. Then the beads were incubated 16 hr at 4°C in a rotor shaker with cleared extract of *E. coli* strains (Rosetta DE3) expressing His‐tagged recombinant proteins. Beads were washed eight times and proteins eluted by incubating beads for 10 min with elution buffer (40 mmol/L Tris pH 8.0, 500 mmol/L NaCl, and 50 mmol/L reduced glutathione). The eluted proteins were resolved by SDS‐PAGE and analyzed by Coomassie blue staining or Western blotting.

### 2.5 Cell invasion assay

Bacteria ability to invade HeLa cells was tested with a gentamicin protection assay (Sansonetti, Ryter, Clerc, Maurelli, & Mounier, 1986). HeLa cells were grown in Dulbecco's modified Eagle medium (DMEM, Lonza), 10% fetal bovine serum (FBS) in a humidified incubator under 5% CO_2_. Briefly, cells were seeded at 1 × 10^5^ cells/well in 24‐well plates 24 hr prior infection. *Shigella* strains were grown at 37°C to mid‐log phase, washed once with PBS, and suspended in DMEM. Bacteria were then centrifuged onto plates (MOI of 100) at 2,000 *g* for 10 min and further incubated 45 min at 37°C. Infected cells were washed three times and incubated 1 hr with gentamicin (50 μg/ml). Finally, cells were lysed with PBS‐Triton 0.1% and intracellular bacteria were diluted and plated on TSB agar Petri dishes for colony‐forming units (cfu) counting. Hela cells invasion was defined as 100% for the wild‐type strain (M90T).

### 2.6 Contact‐mediated hemolysis

The contact‐mediated hemolysis assay was performed as previously described (Blocker et al., 1999). Bacteria from overnight precultures were diluted (OD^600^: 0.05) and grown at 37°C to mid‐log phase, washed once with PBS, and suspended at a concentration of 1,0^10^ bacteria/ml. Horse red blood cells (Oxoid) were centrifuged at 2,000 *g* for 10 min at 4°C and washed twice with cold PBS. Then 50 μl of each sample was mixed in 96‐well flat bottom and centrifuged at 2,000 *g* for 10 min. After 1 hr incubation at 37°C, the reaction was stopped by the addition of 100 μl of cold PBS. Cells were suspended and further centrifuged at 2,000 *g* for 10 min. Optical density of the supernatant was measured at 540 nm. Red blood cells lysis was defined as 100% for the wild‐type strain (M90T).

### 2.7 Transmission Electron Microscopy

Whole bacterial cells were applied to glow discharged carbon‐coated Formvar copper grids. Bacterial cells were negatively stained with 4% ForMol. Observations were done on a Tecnai 10 (FEI) microscope coupled to a Veleta charge‐coupled device (CCD) camera (Olympus iTEM), and images were captured and analyzed using SIS Olympus iTEM software. Whole bacterial cells were applied to glow discharged carbon‐coated Formvar copper grids and negatively stained with 4% Uranyl acetate. Observations were done on a Tecnai 10 (FEI) transmission electron microscope coupled to a Veleta CCD camera (Olympus iTEM), and images were captured and analyzed using SIS Olympus iTEM software. For SEM, samples were fixed overnight at 4°C in glutaraldehyde 2.5%, 0.1 mol/L cacodylate buffer (pH 7.2), and postfixed in OsO_4_ (2%) in the same buffer. After serial dehydration samples were dried at critical point and coated with platinum by standard procedures. Observations were made in a Tecnai FEG ESEM QUANTA 200 (FEI) and images were processed by SIS iTEM (Olympus) software.

## 3 RESULTS

**Figure 1:**
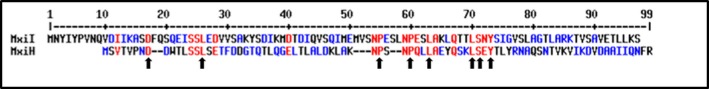
Alignment of MxiI and MxiH proteins from *Shigella flexneri* using Multalin software (Corpet, 1988). Residues mutated in this study are pointed by black vertical arrows. Residues in red are for identity and in blue for similarity

We decided to perform site‐directed mutagenesis within *mxiI* which could result in an *mxiC‐like* mutant phenotype. As MxiI shares 18% of sequence identity with MxiH, which is also implicated in signal transmission, we first generated 8‐point mutations of conserved residues between these two proteins (Figure 1), by replacing them by alanine residues. The mutated variants (generated on pSM6) were introduced in the *mxiI* mutant and the resulting strains were tested for their ability to bind CR on plate, to secrete virulence proteins, to perform contact‐mediated hemolysis (reflecting translocon pore formation), and to invade HeLa cells. As shown in Table 1, six of the eight generated *mxiI* mutants show a phenotype similar to the wild‐type strain for colony color on CR plate, proteins secretion, hemolysis, and cell invasion, one presents exactly the same phenotype as the *mxiI* mutant (*mxiI* ^*L63A*^), and another presents a global reduction in proteins secretion (*mxiI* ^*L26A*^). Nevertheless, none of all the mutations led to a hyper‐red‐colony phenotype on CR plate suggesting that none of them is able to abolish the interaction between MxiI and MxiC.

**Table 1:** General characterization of MxiI mutants

Strains	Colony color	Noninductible secretion	CR induction		% Hemolysis	% Invasion
			Translocators	Effectors		
M90T	Red	+	+	+	100 ± 0.97	100 ± 3.2
*mxi*I	White	−	−	−	0.73 ± 0.56	0 ± 1.2
*mxiI* ^+^	Red	+	+	+	99.25 ± 1.72	104 ± 7.3
*mxiC*	Hyper‐red	+++	Delayed	+	1.17 ± 0.84	9 ± 1.7
mxiI^D17A^	Red	+	+	+	98.5 ± 2.87	95 ± 4.6
mxiI^L26A^	White	−	Reduced	Reduced	7.67 ± 1.84	2 ± 1.9
mxiI^P55A^	Red	+	+	+	135.77 ± 1.22	97 ± 4.6
mxiI^P60A^	Red	+	+	+	103.86 ± 5.08	92 ± 6.4
mxiI^L63A^	White	−	−	−	0.79 ± 0.64	2 ± 2.6
mxiI^Q67A^ a	Pink	+^a^	+^a^	−a	55.41 ± 8.3	76 ± 5.2
mxiI^Q67E^	Pink	+	+	−	65.43 ± 8.9	39 ± 2.9
mxiI^Q67K^	Red	+	+	+	67.16 ± 0.65	41 ± 3.7
mxiI^L70A^	Red	+	+	+	105.24 ± 7.19	96 ± 1.8
mxiI^Y73A^	Red	+	+	+	101.42 ± 2.34	65 ± 7.2
mxiI^S71A^	Red	+	+	+	90.34 ± 10.58	101 ± 2.1
mxiI^T82R^	Hyper‐red	+++	Delayed	+	1.85 ± 1.46	35 ± 3.2
mxiI^T82K^	Hyper‐red	+++	Delayed	+	3.40 ± 1.69	52 ± 8.4
mxiI^T82A^	Red	+	+	+	87.32 ± 8.99	92 ± 6.2
mxiI^T82E^	Red	+	+	+	71.98 ± 9.95	95 ± 5.3

In blank: residues mutated by site‐directed mutagenesis based on the homology between MxiI and MxiH. In grey: residues mutated by random mutagenesis on *mxiI* and harboring a *mxiC‐like* phenotype.

CR, Congo red.

a Cherradi et al. (2013).

### 3.1 The *mxiI* ^*L26A*^ *strain presents a global defect in secretion and is crucial for Spa40 binding*

**Figure 2:**
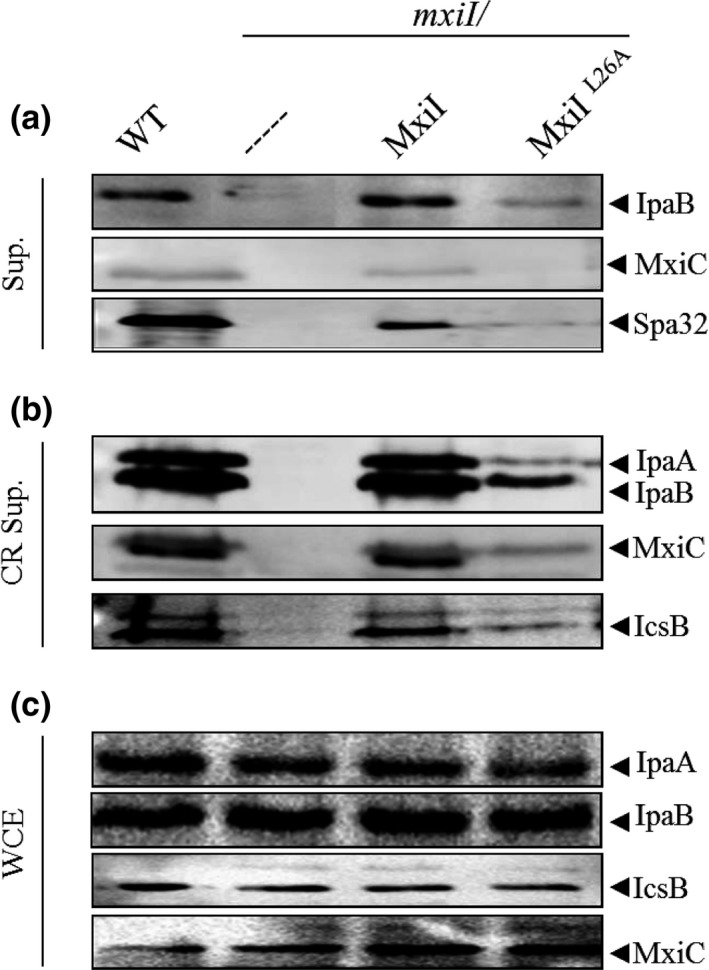
*mxi* *I*^*L*^ ^*26A*^ strain shows a global defect in secretion. Proteins of (a) culture supernatants (Sup.), (b) Congo red‐induced supernatants (CR Sup.), and (c) whole‐cell extracts (WCE) of strains: wild‐type (WT), *mxiI* mutant (*mxiI*), *mxiI* mutant complemented with plasmid‐expressing native MxiI (*mxiI/*MxiI) or its derivative‐expressing MxiI^L^ ^26A^ (*mxiI*/MxiI^L^ ^26A^) were resolved on SDS‐PAGE and analyzed by Western blot using polyclonal antibodies against IpaB, IpaA, MxiC, Spa32, and IcsB. All experiments were performed at least three times

The L26A mutation led to a global decrease in proteins secretion under both constitutive and induced conditions, although proteins were produced like in the wild‐type strain (Figure 2a–c). Indeed, we observed that neither MxiC, nor effectors (IpaA and IcsB) and only a small amount of IpaB were secreted upon CR induction compared to the wild‐type strain (Figure 2b). Interestingly, we also noticed that even Spa32, the needle length regulator, was barely detectable in *mxiI* ^*L26A*^ strain (Figure 2a). This secretion defect of *mxiI* ^*L26A*^ along with its very low performance in hemolysis and invasion assays (Table 1) suggest that this mutation might have affected the two switches in T3S; first, the needle subunits to translocators secretion switch, regulated by Spa40 and Spa32 (Botteaux et al., 2008, 2010), and secondly, the translocators to effectors secretion switch regulated by MxiC (Botteaux et al., 2009; Martinez‐Argudo & Blocker, 2010).

**Figure 3:**
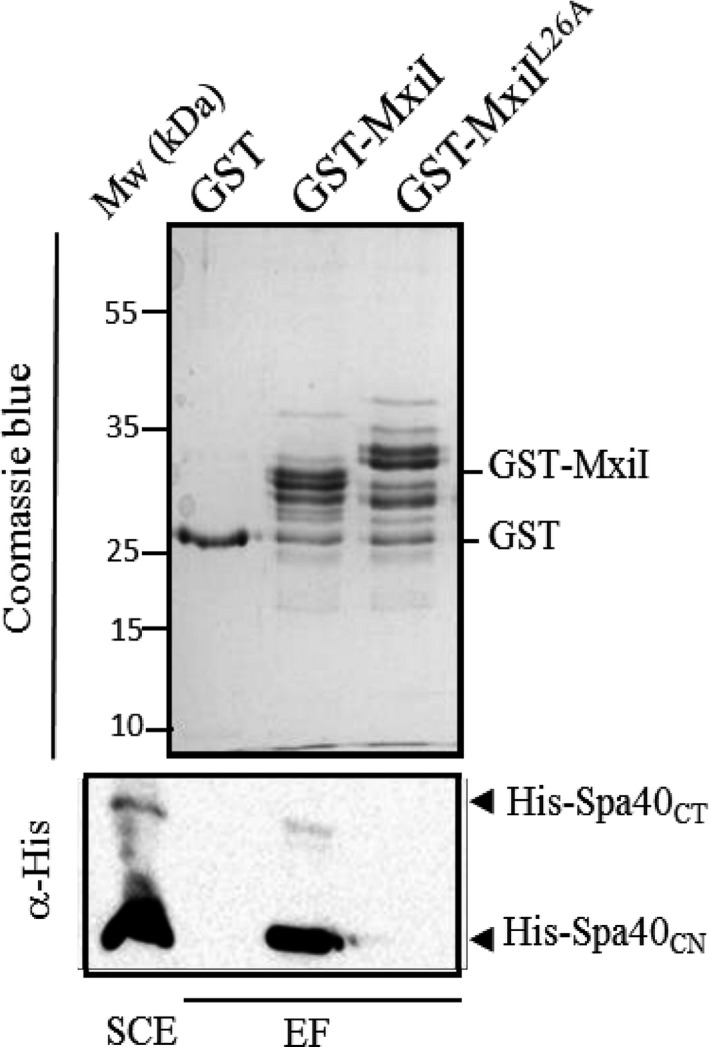
MxiI^L^ ^26A^ does not interact with the cytoplasmic domain of Spa40. Soluble cell extract (SCE) of *E. coli‐*producing His‐Spa40_CT_ was incubated with GST alone, GST‐MxiI, and GST‐MxiI^L^ ^26A^ bound to glutathione‐Sepharose. Eluted fractions (EF) were resolved by SDS‐PAGE and analyzed by Coomassie blue staining or by Western blot using monoclonal antibodies against His‐tag. His‐Spa40_CT_ corresponds to the cytoplasmic part of Spa40 (residues 205–342) and His‐Spa40_CN_ corresponds to the cleaved form (residues 205–258). The binding assay was repeated at least three times

We have previously shown that MxiI interacts with the cytoplasmic domain of Spa40, called Spa40_CT_ (Cherradi et al., 2013). Moreover, it has been shown that MxiI homologues, PrgJ (*Salmonella*) and YscI (*Yersinia*), play a role in substrate specificity switching and functional needles formation (Lefebre & Galán, 2014; Wood, Jin, & Lloyd, 2008). As the *mxiI* ^*L26A*^ mutant shows a global secretion defect but harbors a needle structure (Figure S2), we supposed that this residue might disrupt the switch from needle subunits to translocators secretion by impairing the MxiI–Spa40_CT_ interaction. To test our hypothesis, we generated the GST‐MxiI^L26A^ and performed a GST pull‐down assay with His‐Spa40_CT (205–342)_. In contrast to unmodified GST‐MxiI, GST‐MxiI^L26A^ did not co‐elute His‐Spa40_CT_ nor His‐Spa40_CN (205–258)_ (cleaved form,) even if produced at a similar level (Figure 3). This finding shows that MxiI residue L26 is involved in the interaction between the predicted inner‐rod protein MxiI and the cytoplasmic domain of Spa40 and that this interaction is probably important for proteins secretion but not for needle assembly.

### 3.2 Inactivation of mxiC in the mxiI^L26A^ mutant restores effectors secretion

**Figure 4:**
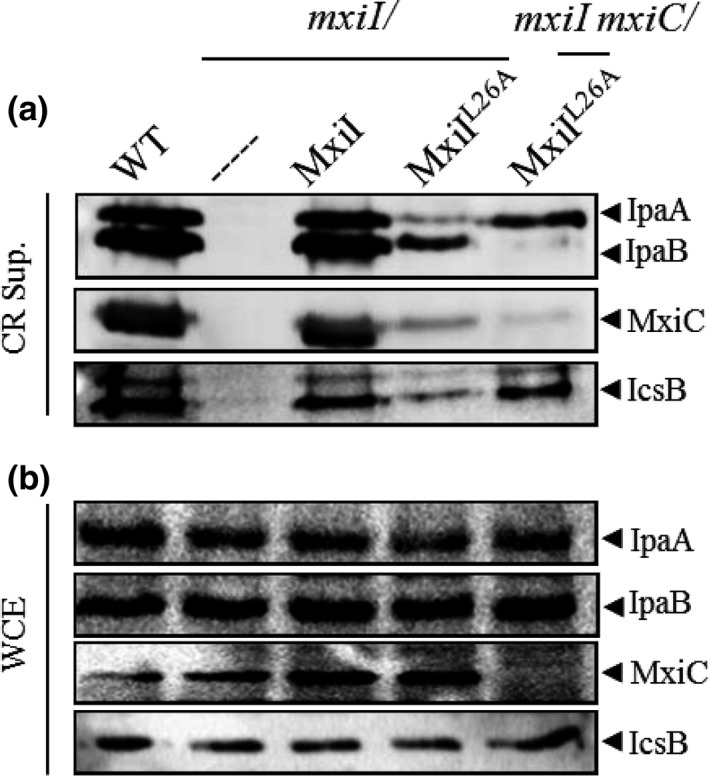
Inactivation of *mxiC* restores effectors secretion in a *mxi* *I*^L^ ^26A^ mutant. Proteins of (a) Congo red supernatants (CR Sup.) or of (b) whole‐cell extracts (WCE) of strains: wild‐type (WT), *mxiI* mutant (*mxiI*), *mxiI* mutant‐expressing MxiI (*mxiI*/MxiI), *mxiI* mutant expressing the variant MxiI^L^ ^26A^ (*mxiI*/MxiI^L^ ^26A^), and the *mxiI mxiC* double mutant‐expressing MxiI^L^ ^26A^ variant (*mxiI mxiC*/MxiI^L^ ^26A^) were analyzed by Western blot using polyclonal antibodies against IpaA, IpaB, MxiC, and IcsB. All experiments were performed at least three times

**Figure 5:**
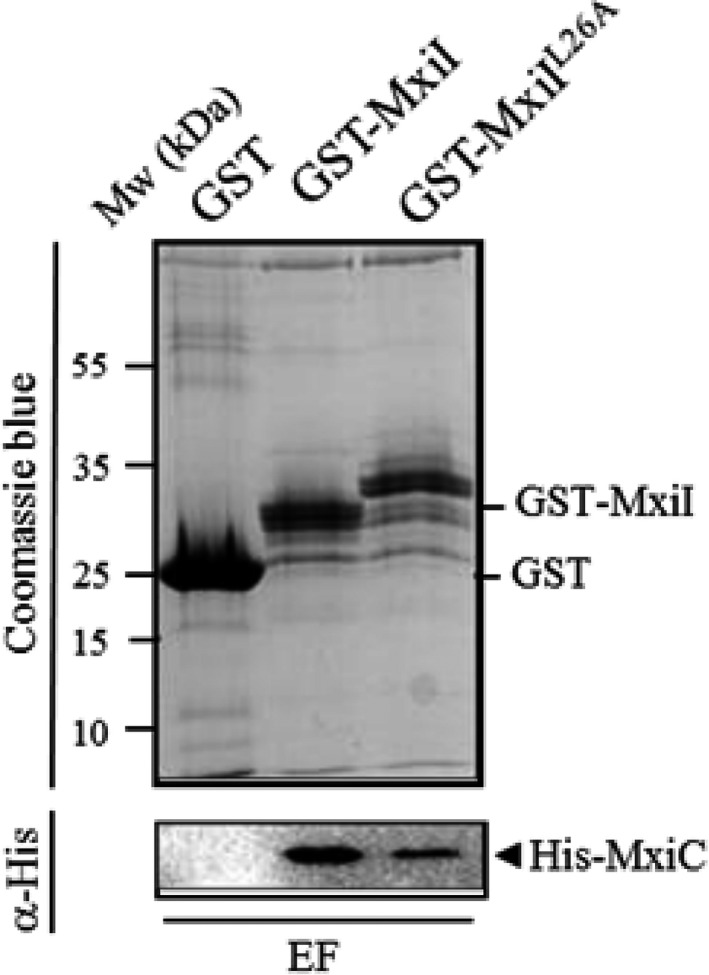
The MxiI^L^ ^26A^ variant interacts with MxiC. Soluble cell extract of *E. coli* producing His‐MxiC was incubated with GST alone, GST‐MxiI, and GST‐MxiI^L^ ^26A^ bound to glutathione‐Sepharose. Eluted fractions (EF) were resolved by SDS‐PAGE and analyzed by Coomassie blue staining or Western blot using monoclonal antibodies against His‐tag. The binding assay was repeated at least three times

As the *mxiI* ^*L26A*^ mutant is not able to secrete neither MxiC nor any effectors in the presence of CR, we hypothesized that MxiC is blocked, sequestering effectors in this mutant. To test this hypothesis, we have expressed the MxiI^L26A^ variant into the *mxiC mxiI* double mutant and observed that, in this background, the variant MxiI^L26A^ allows effectors secretion like in a wild‐type strain (Figure 4a). So, like the previously described MxiI^Q67A^ variant, MxiI^L26A^ cannot promote effectors secretion, maybe due to a lack of MxiC secretion, and this defect is not a consequence of the lack of proteins production (Figure 4b). Quite logically we have tested its capacity to bind MxiC in order to retain it in the bacterial cytoplasm. As shown in Figure 5, GST‐MxiI^L26A^ is still able to bind His‐MxiC confirming our previously proposed model for the MxiI–MxiC complex function (Cherradi et al., 2013).

### 3.3 Mutations of residue T82 lead to a mxiC‐like secretion phenotype

**Figure 6:**
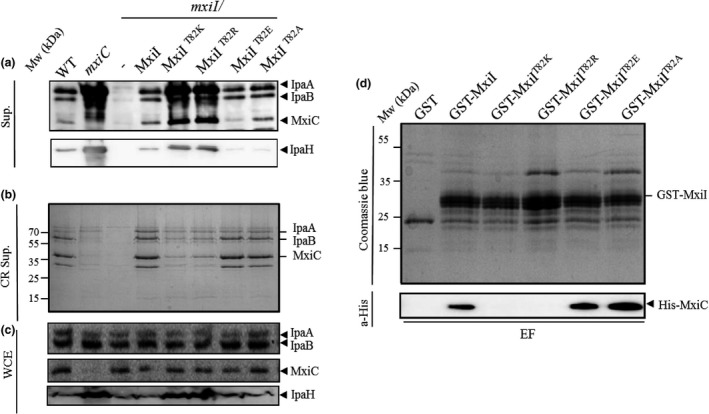
The MxiI residue T82 is crucial for the control of effectors secretion and MxiC binding. Proteins of (a) Culture supernatants (Sup.), (b) Congo red supernatant (CR Sup.), or (c) whole‐cell extracts (WCE) of strains: wild‐type (WT), *mxiC* mutant *(mxiC*)*, mxiI* mutant (*mxiI*), *mxiI* mutant‐expressing MxiI, or its variants MxiI^T^ ^82K^, MxiI^T^ ^82R^, MxiI^T^ ^82E^, and MxiI^T^ ^82A^, were resolved on SDS‐PAGE and analyzed by Coomassie blue staining or by Western blot using polyclonal antibodies against IpaA, IpaB, IpaH, and MxiC. (d) Soluble cells extract of *E. coli‐*producing His‐MxiC was incubated with GST alone, GST‐MxiI, and its derivatives (GST‐MxiI^T^ ^82K^, GST‐MxiI^T^ ^82R^, GST‐MxiI^T^ ^82E^, and GST‐MxiI^T^ ^82A^) bound to glutathione‐Sepharose. Eluted fractions (EF) were resolved by SDS‐PAGE and analyzed by Coomassie blue staining and by Western blot using monoclonal antibodies against His‐tag. All experiments were performed at least three times

As we did not find any mutation within *mxiI* producing an *mxiC*‐like phenotype by site‐directed mutagenesis, we decided to perform random mutagenesis on *mxiI*. We have created a library of *mxiI* mutants by error‐prone PCR on the pSU18‐*mxiI*. After transforming this library into the *mxiI* mutant, we have screened the resulting strains for their CR‐binding properties. All strains harboring a hyper‐red phenotype on CR plates (like previously shown for the *mxiC* mutant) were subsequently analyzed for their constitutive secretion phenotype. Two of them, harboring a mutation of the T82 residue, into a lysine (T82K) or an arginine (T82R), presented the same phenotype than the *mxiC* mutant as they constitutively secrete early and late effectors (Figure 6a). We also observed that MxiC was prematurely (i.e. before induction) secreted by these two strains compared to the wild type (Figure 6a). To confirm the *mxiC*‐like phenotype, we have also tested their ability to secrete translocators under induced conditions. As shown in Figure 6b, the two mutants secrete effectors at a level similar to the wild‐type strain but present a delay in translocators secretion as described for the *mxiC* mutant (Botteaux et al., 2009). This result clearly shows that MxiI residue T82 is important for the control of the timing of MxiC secretion and the subsequent translocators and effectors secretion. The Figure 6c shows that the observed effect on secretion was not due to the lack of proteins production. Both variants of MxiI also present the same defect in hemolysis than the *mxiC* mutant even if they are able to enter cells more efficiently than the *mxiC* mutant (Table 1).

### 3.4 The MxiI T82 residue is crucial for MxiC binding

We have previously shown that MxiI interacts with MxiC and that this interaction is crucial for the transmission of the activation signal and for effectors sequestration inside bacteria prior to T3S induction (Cherradi et al., 2013). MxiI^T82R^ and MxiI^T82K^ variants induce a secretion phenotype similar to that of a *mxiC* mutant, suggesting that MxiI interaction with MxiC might have been abolished by these mutations. So, we generated the mutations T82R and T82K on the plasmid‐encoding GST‐MxiI and performed GST pull‐down assay. A soluble extract of an *E. coli* strain‐producing His‐MxiC was incubated with GST‐MxiI, as a positive control, GST‐MxiI derivatives or GST alone, previously bound on glutathione‐Sepharose beads. Proteins retained on the beads were eluted with glutathione and Western blot analysis of eluted proteins indicated that His‐MxiC does not interact anymore with GST‐MxiI^T82R^ and GST‐MxiI^T82K^ (Figure 6d). These results were confirmed using plasmid‐expressing MxiI fused to a His‐tag (pET30a‐*mxiI*) and a GST‐MxiC (Figure S3). Our results show that the MxiI residue T82 is crucial for MxiC binding and confirm that the observed *mxiC‐*like mutant phenotype is due to the loss of MxiI–MxiC complex formation.

### 3.5 Charge of the residue T82 is involved in secretion control

As both random mutations leading to an *mxiC‐*like phenotype are replacements of the noncharged threonine residue by positively charged ones (lysine and arginine), we thought that charge of the residue could influence its capacity to bind MxiC and to control secretion. To answer this question, we have replaced the T82 residue by a negatively charged (glutamate) and a hydrophobic nonpolar (alanine) residue on the pSM6. The proteins secreted by the different *mxiI* mutants (expressing MxiI^T82E^ and MxiI^T82A^) under both constitutive and induced conditions were analyzed. Unlike *mxiI* ^*T82K*^ and *mxiI* ^*T82R*^ strains, *mxiI* ^*T82E*^ and *mxiI* ^*T82A*^ strains allow proteins secretion under induced and noninduced conditions like the wild‐type strain (Figure 6a,b). As expected, both variants were still able to bind MxiC (Figure 6d). These results confirm that the charge of MxiI T82 residue is crucial for MxiC binding and secretion and strengthen the role of the MxiI–MxiC interaction in effectors secretion control.

### 3.6 Charge of the residue Q67 influences the secretion signal transmission

**Figure 7:**
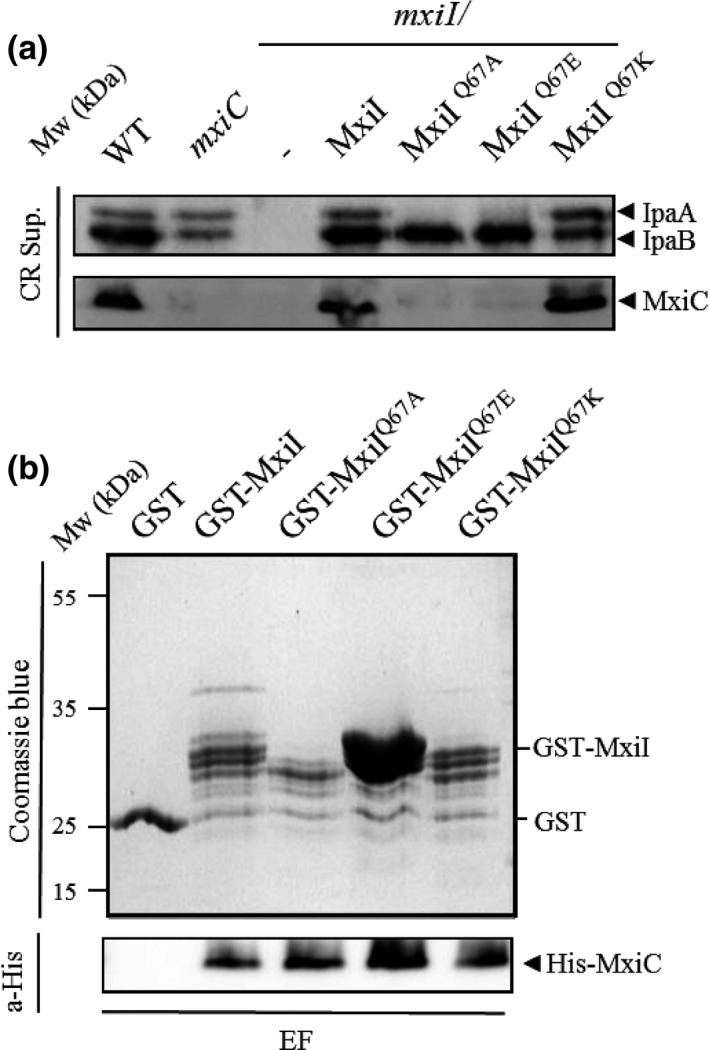
The charge of the MxiI residue Q67 is involved in effectors secretion control. (a) Proteins of CR supernatant of strains: wild‐type M90T (WT), *mxiC* mutant *(mxiC*)*, mxiI* mutant (*mxiI*), *mxiI*/pSM6 (expressing MxiI), and its variants expressing MxiI^Q^ ^67A^, MxiI^Q^ ^67E^, and MxiI^Q^ ^67K^ were analyzed by SDS‐PAGE and by Western blot using antibodies against IpaA, IpaB, and MxiC. (b) Soluble cell extract of *E. coli‐*producing His‐MxiC was incubated with GST alone, GST‐MxiI, and its derivatives bound to glutathione‐Sepharose. Eluted fractions (EF) were resolved by SDS‐PAGE and analyzed by Coomassie blue staining and Western blot using monoclonal antibodies against His‐tag. All experiments were performed at least three times

As we have shown the importance of the residue charge in MxiI function, we decided to mutate the Q67 residue, known to block effectors secretion upon T3SS activation when replaced by an alanine residue (Cherradi et al., 2013), into a negatively charged residue (*mxiI* ^*Q67E*^) or a positively charged one (*mxiI* ^*Q67K*^). We found that the *mxiI* ^*Q67E*^, like the *mxiI* ^*Q67A*^, presents an “effector mutant” phenotype while the *mxiI* ^*Q67K*^ allows proteins secretion like the wild‐type strain (Figure 7a). Nevertheless, all these variants present a defect in hemolysis and invasion independently of their secretion profiles (Table 1). Not surprisingly, these variants still interact with MxiC (Figure 7b).

### 3.7 The residues 74–97 of MxiI are responsible for the interaction with MxiC

**Figure 8:**
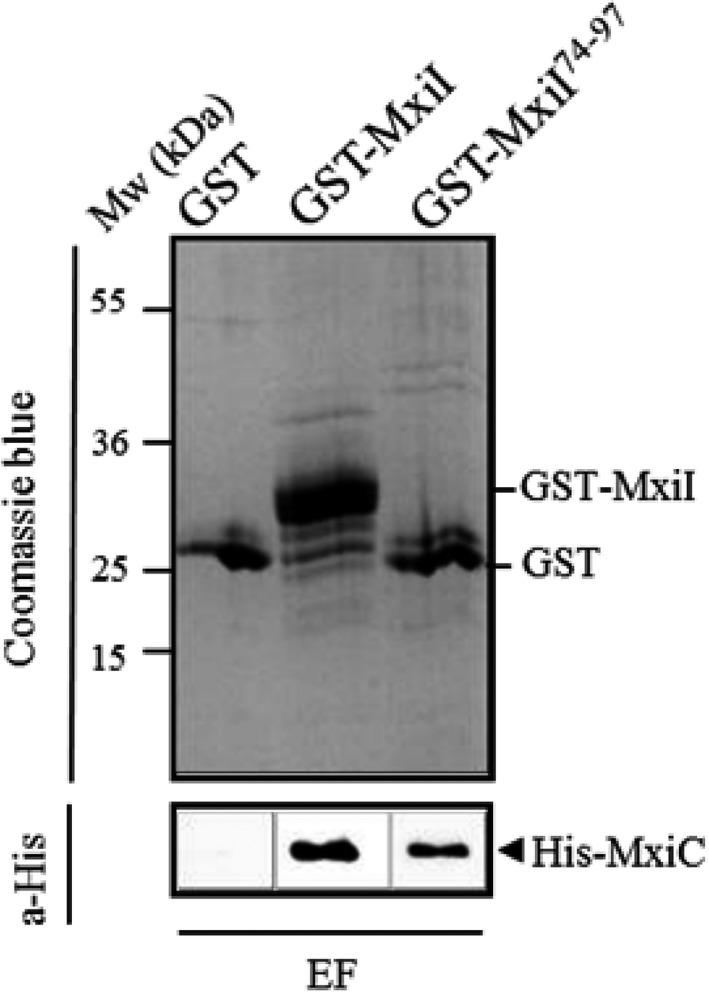
The C‐terminal residues 74 –97 of MxiI are sufficient for MxiC binding. Soluble cell extract of *E. coli‐*producing His‐MxiC was incubated with GST alone, GST‐MxiI, and GST‐MxiI^74–97^ bound to glutathione‐Sepharose. Eluted fractions (EF) were resolved by SDS‐PAGE and analyzed by Coomassie blue staining and Western blot using monoclonal antibodies against His‐tag. The binding assay was repeated at least three times

We have previously shown that the interaction between MxiC and MxiI is conserved among T3SSs (Cherradi et al., 2013). As sequences alignment between MxiI homologous proteins highlights the high level of conservation in the C‐terminal part of MxiI (Figure S1A) and as the T82 residue seems crucial for the MxiC binding, we thought that this domain could be directly involved in the interaction with MxiC. Moreover, the in silico modeling (on Swiss model server) using MxiH as a template (Figure S1B) allows the alignment of T82 residue on MxiH N65 which faces the needle lumen. We therefore assumed that the putative C‐terminal helix of MxiI is probably lining the needle lumen like for MxiH (Demers et al., 2013; Verasdonck et al., 2015). To test whether residues 74–97 of MxiI are sufficient for MxiC binding, we constructed plasmid pGEX4T1‐*mxiI* ^*74–97*^ expressing the C‐terminal domain of MxiI in fusion to GST and performed a GST pull‐down assay. We revealed an interaction between MxiC and the MxiI^74–97^ (Figure 8) confirming that the C‐terminal domain of MxiI, corresponding to residues 74 – 97, is sufficient for MxiC binding.

## 4 DISCUSSION

We have previously proposed that MxiC bound to MxiI could prevent effectors secretion by forming a complex docked at the T3SA entry gate (Cherradi et al., 2013). Indeed, mutation in MxiC that abolishes MxiI interaction (MxiC^F206S^) leads to a *mxiC*‐mutant phenotype in terms of early effectors secretion (Cherradi et al., 2013). Nevertheless, this mutation still allows CR response as a wild‐type strain suggesting that the two functions of MxiC can be uncoupled, as confirmed recently (Roehrich et al., 2016). To strengthen our model, we have looked for a point mutation in MxiI that, by losing MxiC binding capacities, will also lead to the loss of effectors secretion control (*mxiC*‐like phenotype). To do so, we have first mutated conserved residues between MxiI and MxiH as the latter is also implicated in signal transmission (Kenjale et al., 2005; Martinez‐Argudo & Blocker, 2010). As this approach failed to provide us the desired phenotype, we have generated random mutations and found two mutants (*mxiI* ^*T82K*^ and *mxiI* ^*T82R*^) harboring an *mxiC*‐like phenotype (hyper‐red colonies). As expected these mutants were no longer able to interact with MxiC, supporting our initial hypothesis. Moreover, the loss of MxiC–MxiI interaction leads to an earlier secretion of MxiC (before induction) and explains why MxiC is no longer able to ensure its role in promoting translocators secretion in these backgrounds. Indeed, unlike the MxiC^F206S^ variant, the MxiI^T82K^ and MxiI^T82R^ show the same defect in translocators secretion after CR induction than the *mxiC* mutant. Interestingly, the T82 residue is conserved with PrgJ, the MxiI homologue from *Salmonella,* which can interact with the MxiC counterpart, InvE (Cherradi et al., 2013). Thus, at this stage, we can postulate that MxiI, by interacting with MxiC, acts as a timer for its secretion and that MxiC secretion serves as a signal to secrete effectors, function that could be conserved between T3SSs. Solid‐state NMR showed that the N‐terminal part of MxiH lies on the outside face of the needle while the C‐terminal part (the most conserved one among MxiH homologous proteins in other T3SSs) is lining the lumen (Demers et al., 2013; Verasdonck et al., 2015). Interestingly, based on sequence and structure homology with MxiH, we assumed that the residue T82 (corresponding to N65 in MxiH) could be exposed in the lumen of the inner rod and thus be directly involved in MxiC binding. Moreover, we previously showed that the MxiC–MxiI interaction is conserved among other T3SSs (Cherradi et al., 2013), and we know that the C‐terminal part of MxiI is the most conserved among homologous proteins (Figure S1A). In light of this, we have cloned the last 23 residues of MxiI (MxiI^74–97^) and shown that they are effectively sufficient to bind MxiC, supporting the conservation of the regulatory mechanism. As the C‐terminal part of MxiI harboring the T82 residue is not well conserved with MxiH (Figure 1), it could explain why MxiC is able to bind specifically to MxiI and not to MxiH as previously shown (Cherradi et al., 2013). The existence of a complex between MxiC and MxiI was shown using copurification assays in which MxiC is probably folded (Deane, Roversi, King, Johnson, & Lea, 2008b) and MxiI disordered (Zhong et al., 2012). These experimental conditions could seem far away from the conditions encountered at the base of the needle, where MxiC is probably unfolded to be secreted and MxiI folded in the needle structure. Nevertheless, MxiC even in a folded state is an elongated rod‐shaped molecule, mainly composed of α‐helices, providing the maximal exposure of surface area and considerable binding interfaces. In the light of the inner diameter of the needle (Radics, Königsmaier, & Marlovits, 2014), it is tempting to speculate that the helices are still presents when MxiC is secreted by T3SS and that they could be responsible of the MxiI binding. On the other hand, we have shown that the domain of MxiI interacting with MxiC is located inside its last C‐terminal helix, which was shown for PrgJ to be ordered, even in solution (Zhong et al., 2012).

As the electrostatic surface of some effectors is negatively charged, Rathinavelan et al. (2010) proposed that repulsive forces between secreted proteins and the internal face of the channel could facilitate the transit into the needle. Although this model is based on a wrong orientation of the needle subunits (Verasdonck et al., 2015), we showed here that the charge of the residues on MxiI seems implicated in its function. Indeed, replacement of T82 by positively charged residues (lysine or arginine) leads to an *mxiC*‐like phenotype and the wild‐type phenotype can be restored by changing into a neutral (A) or negative (E) residues. Moreover, the charge seems to impact directly MxiC binding as MxiI^T82K^ and MxiI^T82R^ totally abolish MxiC binding. Thus, we can postulate that this deregulated phenotype is due to the loss of interaction between the MxiI variants and MxiC which results in MxiC early secretion. Interestingly, the change in negative charges on the surface‐exposed residues of MxiC by positive ones also leads to a deregulated secretion phenotype by an unexplained mechanism (Roehrich, Guillossou, Blocker, & Martinez‐Argudo, 2013) that could be due to a loss of interaction with MxiI.

The same effect was observed for the Q67 residue which can lead to an “effector mutant” phenotype when replaced by an alanine or by a glutamic acid or to a phenotype similar to the wild‐type strain when mutated into a lysine residue. Based on the MxiH homology, this residue could be involved in the MxiI monomers lateral contact to form the inner rod, rather than lining the lumen. Furthermore, all these variants are still able to bind MxiC. Thus, like *yscI* ^*Q84A*^ or *prgJ* ^*Q71A*^, homologous to Q67 residue of MxiI in *Yersinia* or *Salmonella,* respectively, (Figure S1A), we could think that this mutant presents some defect in inner‐rod assembly (Lefebre & Galán, 2014; Wood et al., 2008). Even not conserved, the mutation of the K69 residue within MxiH leads exactly to the same phenotype than *mxiI* ^*Q67A/E*^ (effectors mutant) and is rescued by *mxiC* inactivation, but the *mxiH* ^*K69A*^ needles are shorter than the wild‐type strain (Kenjale et al., 2005; Martinez‐Argudo & Blocker, 2010). Taken this into account, structural changes in the needle could explain the defect in hemolysis that we observed with these mutants, even in the *mxiI* ^*Q67K*^ which secretes all the proteins at a level similar to the one of a wild‐type strain. Further precise structural studies are needed to investigate this hypothesis.

In the course of our study, we also found two mutations within MxiI (MxiI^L26A^ and MxiI^L63A^) that decrease, even abolish, translocators and effectors secretion. As we failed to detect the MxiI variants in *Shigella* background by Western blot, we cannot exclude a lack of expression in the *mxiI* ^*L63A*^ strain explaining the absence of the needle, especially given that the MxiI^L63A^ counterpart in *Yersinia* (YscI^L80A^) is not expressed (Wood et al., 2008). As the *mxiI* ^*L26A*^ assembles needles, we have tried to restore effectors secretion by inactivating *mxiC* and showed that the lack of effectors secretion in this strain was due to a sequestration of MxiC inside the bacteria. To explain the defect in translocators secretion in this strain, we studied the impact of this mutation on its interaction with the cytoplasmic part of Spa40 (Spa40_CT_). Indeed Spa40_CT_ is known to control substrate specificity switch between needle components and translocators secretion. Interestingly MxiI^L26A^ is no longer able to interact with Spa40_CT_ and Spa32 seems weakly secreted. So, as previously shown for MxiI homologous proteins (Marlovits et al., 2006; Wood et al., 2008), MxiI seems to have a role in the substrates switching process. This lack of translocators secretion is also observed in a *Salmonella* strain‐expressing PrgJ^L29A^ but InvJ, the Spa32 counterpart in *Salmonella,* is secreted and the needle complexes are similar to wild‐type ones in this strain (Lefebre & Galán, 2014).

The results presented here strengthened our previous model in which the MxiC–MxiI complex regulates the effectors secretion. In fact, we have shown that point mutation in *mxiI* can lead to the same phenotype than the *mxiC* mutant by impairing their mutual interaction. The domain responsible for this interaction was also identified and its localization in a highly conserved domain within MxiI homologous proteins suggests that this mechanism is probably conserved among others T3SSs. Nevertheless, further structural and electrostatic studies of the inner rod would allow a better understanding of the mechanism of signal transmission through the T3SS needle.

## CONFLICT OF INTEREST

None declared.
