# Supplementary figures and images for: The inner‐rod component of Shigella flexneri type 3 secretion system, MxiI, is involved in the transmission of the secretion activation signal by its interaction with MxiC
Source: Microbiologyopen. 2017 Dec 1;7(1):e00520. doi: 10.1002/mbo3.520 (PMC5822323; doi:10.1002/mbo3.520)

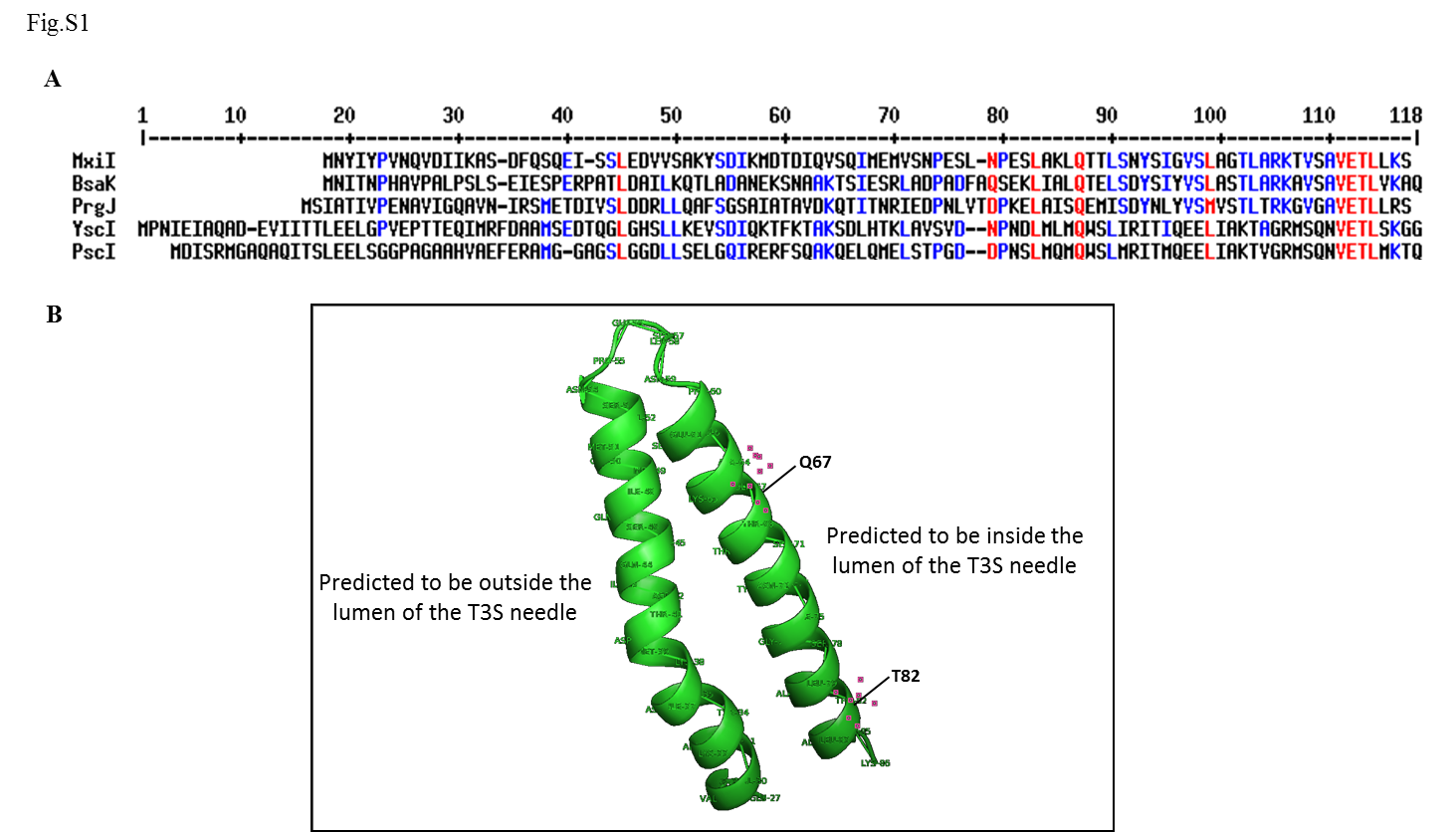

Supplement: Supplementary file 1 [file MBO3-7-na-s001.tif]

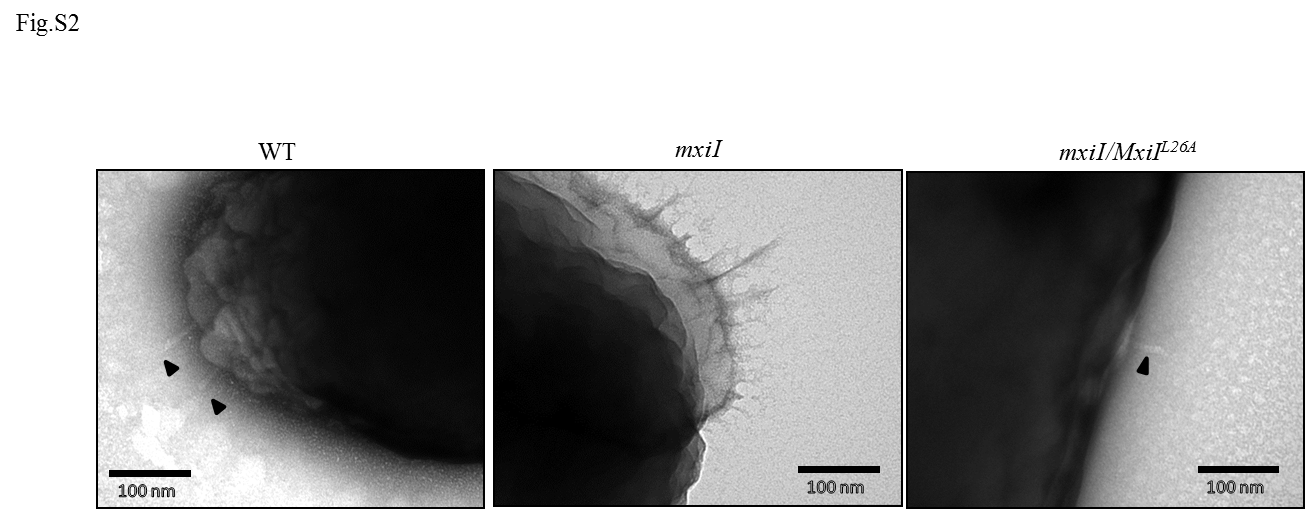

Supplement: Supplementary file 2 [file MBO3-7-na-s002.tif]

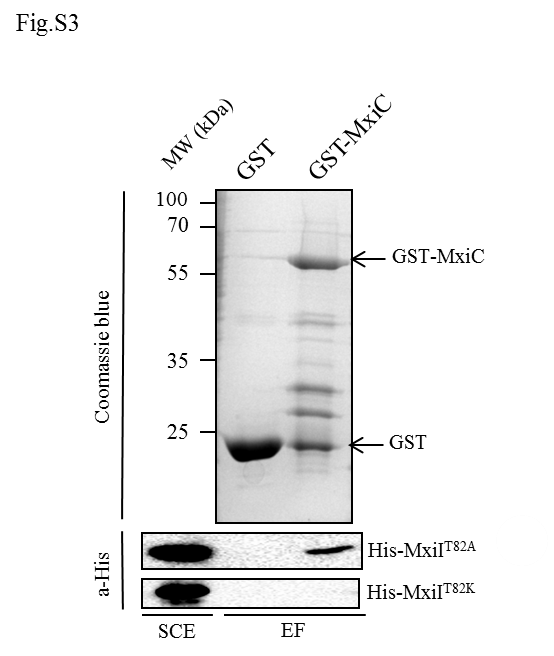

Supplement: Supplementary file 3 [file MBO3-7-na-s003.tif]
