# Supplementary material for: The inner‐rod component of Shigella flexneri type 3 secretion system, MxiI, is involved in the transmission of the secretion activation signal by its interaction with MxiC
Source: Microbiologyopen. 2017 Dec 1;7(1):e00520. doi: 10.1002/mbo3.520 (PMC5822323; doi:10.1002/mbo3.520)
Supplement: Supplementary file 4 [file MBO3-7-na-s004.docx]

**Supplementary data**

| **Strains** | **Features** | **Reference** |
| --- | --- | --- |
| M90T | Wild type *Shigella flexneri* serotype5 SmR | (Allaoui *et al.*, 1992) |
| *mxiC* | *mxiC*::*ble* mutant | (Botteaux *et al.*, 2009) |
| *mxiI* | *mxiI*::*aphA*-3 mutant | (Blocker *et al.*, 2001) |
| *mxiC mxiI* | *mxiC*::*ble mxiI*::*aphA*-3 double mutant | (Cherradi *et al.,* 2013) |
| **Plasmids** |  |  |
| pET30a (+) | Expression vector for His tag fusion | Novagen |
| pGEX4T1 | Expression vector for GST fusion | GE Healthcare |
| pSU18 | Low copy expression vector | (Bartolome et al., 1991) |
| pSM6 | pSU18-*mxiI* | (Cherradi et al., 2013) |
| pSM22 | pSU18-*mxiID17A* | This study |
| pSM103 | pSU18-*mxiII23A* | This study |
| pSM32 | pSU18-*mxiIL26A* | This study |
| pSM19 | pSU18-*mxiIP55A* | This study |
| pSM58 | pSU18-*mxiIP60A* | This study |
| pNE64 | pSU18-*mxiIL63A* | This study |
| pSM33 | pSU18-*mxiIQ67A* | (Cherradi et al., 2013) |
| pSM104 | pSU18-*mxiIQ67E* | This study |
| pSM105 | pSU18-*mxiIQ67K* | This study |
| pSM59 | pSU18-*mxiIL70A* | This study |
| pSM21 | pSU18-*mxiIS71A* | This study |
| pJH1 | pSU18-*mxiIY73A* | This study |
| pSM62 | pSU18-*mxiIT82R* | This study |
| pSM80 | pSU18-*mxiIT82K* | This study |
| pSM79 | pSU18-*mxiIT82E* | This study |
| pSM66 | pSU18-*mxiIT82A* | This study |
| pNE65 | pSU18-*mxiIV91A* | This study |
| pNE66 | pSU18-*mxiIL94A* | This study |
| pSL24 | pGEX4T1-*mxiI* (GST-MxiI) | (Cherradi et al., 2013) |
| pSM37 | pGEX4T1-*mxiIQ67A* (GST- MxiIQ67A) | (Cherradi et al., 2013) |
| pNE68 | pGEX4T1-*mxiIQ67E* (GST- MxiIQ67E) | This study |
| pNE69 | pGEX4T1-*mxiIQ67K* (GST- MxiIQ67K) | This study |
| pNE46 | pGEX4T1-*mxiIT82R* (GST- MxiIT82R) | This study |
| pNE47 | pGEX4T1-*mxiIT82K* (GST- MxiIT82K) | This study |
| pNE48 | pGEX4T1-*mxiIT82E* (GST- MxiIT82E) | This study |
| pNE49 | pGEX4T1-*mxiIT82A* (GST- MxiIT82A) | This study |
| pNE45 | pGEX4T1-*mxiIL26A* (GST- MxiIL26A) | This study |
| pNE67 | pGEX4T1-*mxiIL63A* (GST- MxiIL63A) | This study |
| pNE63 | pGEX4T1-*mxiI_74-97_* (GST- MxiI74-97) | This study |
| pSL30 | pET30a(+)-*mxiC* (His-MxiC) | (Cherradi et al., 2013) |
| pMC1 | pQE30-*spa40_CT_* (His-spa40_CT_) | (Botteaux et al., 2008) |

**Table S1: Plasmids and strains used in this study**

| **Primer** | **Sequence 5’ – 3’** | **Restriction site** |
| --- | --- | --- |
| MxiI-74-97s | GTC-GGA-TCC-TCA-ATA-GGA-GTA-TCA-TTA-GCT-G |  |
| MxiI-D17Aas | TTG-AGA-TTG-AAA-AGC-GCT-AGC-TTT-GAT-AAT | *Afe*I |
| MxiI-D17As | ATT-ATC-AAA-GCT-AGC-GCT-TTT-CAA-TCT-CAA | *Afe*I |
| MxiI-L26Aas | GAC-GTC-TTC-TGC-AGA-TGA-TAT-CTC-TTG | *Pst*I |
| MxiI-L26As | CAA-GAG-ATA-TCA-TCT-GCA-GAA-GAC-GTC | *Pst*I |
| MxiI-P55Aas | TAA-TGA-TTC-TGC-ATT-CGA-AAC-CAT-CTC-CAT | *Bst*BI |
| MxiI-P55As | ATG-GAG-ATG-GTT-TCG-AAT-GCA-GAA-TCA-TTA | *Bst*BI |
| MxiI-P60Aas | TAA-CTT-GGC-CAA-GCT-TTC-TGC-GTT-TAA-TGA-TTC-TGG | *Hind*III |
| MxiI-P60As | CCA-GAA-TCA-TTA-AAC-GCA-GAA-AGC-TTG-GCC-AAG-TTA | *Hind*III |
| MxiI-L63Aas | CTG-TAA-CTT-GGC-TGC-AGA-TTC-TGG-GTT-TAA-TGA | *Pst*I |
| MxiI-L63As | AAC-CCA-GAA-TCT-GCA-GCC-AAG-TTA-CAG-ACG-ACG | *Pst*I |
| MxiI-Q67Eas | CGT-CGT-CTC-GAG-CTT-GGC-CAA-AGA | *Xho*I |
| MxiI-Q67Es | GCC-AAG-CTC-GAG-ACG-ACG-CTC-TCA | *Xho*I |
| MxiI-L70Aas | TCC-TAT-TGA-ATA-ATT-CGA-AGC-CGT-CGT-CTG-TAA | *Bst*BI |
| MxiI-L70As | TTA-CAG-ACG-ACG-GCT-TCG-AAT-TAT-TCA-ATA-GGA | *Bst*BI |
| MxiI-S71Aas | TAT-TGA-ATA-ATT-AGC-TAG-CGT-CGT-CTG | *Nhe*I |
| MxiI-S71As | CAG-ACG-ACG-CTA-GCT-AAT-TAT-TCA-ATA | *Nhe*I |
| MxiI-Y73Aas | TGA-TAC-TCC-TAT-CGA-TGC-ATT-TGA-GAG-CGT-CGT-CTG | *Hind*III |
| MxiI-Y73As | ACG-CTC-TCA-AAT-GCA-TCG-ATA-GGA-GTA-TCA-TTA-GCT | *Hind*III |
| MxiI-T82Kas | TTT-TCT-TGC-TAG-CTT-GCC-AGC-TAA-TGA | *Nhe*I |
| MxiI-T82Ks | TCA-TTA-GCT-GGC-AAG-CTA-GCA-AGA-AAA | *Nhe*I |
| MxiI-T82Eas | TTT-TCT-TGC-TAG-CTC-GCC-AGC-TAA-TGA | *Nhe*I |
| MxiI-T82Es | TCA-TTA-GCT-GGC-GAG-CTA-GCA-AGA-AAA | *Nhe*I |
| MxiI-T82Aas | TTT-TCT-TGC-TAA-GGC-GCC-AGC-TAA-TGA | *Hae*II |
| MxiI-T82As | TCA-TTA-GCT-GGC-GCC-TTA-GCA-AGA-AAA | *Hae*II |
| MxiI-V91Aas | AGA-CTT-TAA-AGT-TTC-TGC-AGC-CGA-AAC-TGT | *Pst*I |
| MxiI-V91As | AGA-AAA-ACA-GTT-TCG-GCT-GCA-GAA-ACT-TTA-TTA | *Pst*I |
| MxiI-L94Aas | CTT-TAA-TTA-AGA-CTT-TAA-AGC-AGT-TTC-AAC-AGC | *Dra*I |
| MxiI-L94As | GTT-TCG-GCT-GTT-GAA-ACT-GCT-TTA-AAG-TCT-TAA | *Dra*I |

**Table S2: List of primers used in this study.** Introduced restriction sites are underlined.
